# Identifying a signature of inflammatory blood proteins predicting dementia

**DOI:** 10.1002/alz70856_097996

**Published:** 2025-12-24

**Authors:** Dorsa Abdolkarimi, Yue Liu, Sheena Waters, Sara Calhas, Charles R Marshall, Petroula Proitsi

**Affiliations:** ^1^ Wolfson Institute of Populational Heath, Queen Mary University of London, London, Greater London, United Kingdom; ^2^ Queen Mary University of London, London, Greater London, United Kingdom; ^3^ Centre for Preventive Neurology, Wolfson Institute of Population Health, Queen Mary University of London, United Kingdom, London, London, United Kingdom; ^4^ Centre for Preventive Neurology, Wolfson Institute of Population Health, Queen Mary University of London, London, United Kingdom

## Abstract

**Background:**

Inflammation, driven by systemic mediators, plays a key role in dementia. However, the link between circulating inflammatory proteins and dementia risk remains understudied. We aimed to identify individual inflammatory proteins and an inflammatory proteomics signature (ProSig) associated with incident dementia; investigate the links between ProSig and its proteins with brain image‐derived endophenotypes; and finally explore whether these proteins mediate the effect of known genetic and modifiable risk‐factors with dementia and whether any of these associations were causal.

**Method:**

We leveraged proteomics data from 43,685 participants from the UK Biobank. Cox proportional‐hazard (Cox‐PH) models were used to reveal associations between 728 Olink inflammatory proteins and incident dementia in four sequentially adjusted models. We applied Cox‐PH with LASSO regularisation for feature selection to calculate ProSig, based on the highly associated proteins, predicting incident dementia. Linear regression models investigated associations between ProSig or ProSig proteins and brain endophenotypes in 4,106. Formal mediation analyses investigated whether ProSig or proteins within mediated the association of the APOE‐ε4 genotype, an AD polygenic risk‐score or modifiable risk‐factors with dementia. Mendelian randomisation (MR) analyses assessed causal associations between the ProSig proteins and AD.

**Result:**

We identified up to 218 proteins individually associated with dementia in Cox‐PH models (PFDR< 0.05). A signature of twenty proteins improved the prediction of dementia beyond modifiable risk factors (C‐index=0.84 vs C‐index=0.86, *p* = 6.85x10^‐5^); although it only marginally improved the prediction of incident dementia in a full model including modifiable risk factors and the APOE‐ε4 genotype (C‐index=0.89 vs C‐index=0.90, *p* = 0.11). Three of the ProSig proteins were associated with subcortical brain volume endophenotypes. One of these proteins, involved in neuroinflammation and vascular function, was associated with both dementia and reduced volumes. Mediation analysis indicated that three ProSig proteins mediated the effects of modifiable risk‐factors with dementia. MR analyses confirmed these findings.

**Conclusion:**

Our findings underscore the critical role of inflammation in dementia and emphasise the importance of identifying inflammatory biomarkers for early dementia detection. Findings from our study deepen our understanding of early pathological inflammatory mechanisms and can ultimately enhance recruitment for clinical trials and support the development of personalised therapeutic interventions.

